# Circulating Extracellular Vesicles with Specific Proteome and Liver MicroRNAs Are Potential Biomarkers for Liver Injury in Experimental Fatty Liver Disease

**DOI:** 10.1371/journal.pone.0113651

**Published:** 2014-12-03

**Authors:** Davide Povero, Akiko Eguchi, Hongying Li, Casey D. Johnson, Bettina G. Papouchado, Alexander Wree, Karen Messer, Ariel E. Feldstein

**Affiliations:** 1 Department of Pediatrics, University of California San Diego, La Jolla, California, United States of America; 2 Department of Biostatistics/Bioinformatics - Moores Cancer Center, University of California San Diego, La Jolla, California, United States of America; 3 Department of Pathology, VA San Diego Healthcare System, San Diego, California, United States of America; Institute of Medical Research A Lanari-IDIM, University of Buenos Aires-National Council of Scientific and Technological Research (CONICET), Argentina

## Abstract

**Background & Aim:**

Nonalcoholic fatty liver disease (NAFLD) is the most common chronic liver disease in both adult and children. Currently there are no reliable methods to determine disease severity, monitor disease progression, or efficacy of therapy, other than an invasive liver biopsy.

**Design:**

Choline Deficient L-Amino Acid (CDAA) and high fat diets were used as physiologically relevant mouse models of NAFLD. Circulating extracellular vesicles were isolated, fully characterized by proteomics and molecular analyses and compared to control groups. Liver-related microRNAs were isolated from purified extracellular vesicles and liver specimens.

**Results:**

We observed statistically significant differences in the level of extracellular vesicles (EVs) in liver and blood between two control groups and NAFLD animals. Time-course studies showed that EV levels increase early during disease development and reflect changes in liver histolopathology. EV levels correlated with hepatocyte cell death (r^2^ = 0.64, p<0.05), fibrosis (r^2^ = 0.66, p<0.05) and pathological angiogenesis (r^2^ = 0.71, p<0.05). Extensive characterization of blood EVs identified both microparticles (MPs) and exosomes (EXO) present in blood of NAFLD animals. Proteomic analysis of blood EVs detected various differentially expressed proteins in NAFLD versus control animals. Moreover, unsupervised hierarchical clustering identified a signature that allowed for discrimination between NAFLD and controls. Finally, the liver appears to be an important source of circulating EVs in NAFLD animals as evidenced by the enrichment in blood with miR-122 and 192 - two microRNAs previously described in chronic liver diseases, coupled with a corresponding decrease in expression of these microRNAs in the liver.

**Conclusions:**

These findings suggest a potential for using specific circulating EVs as sensitive and specific biomarkers for the noninvasive diagnosis and monitoring of NAFLD.

## Introduction

Non-alcoholic Fatty Liver Disease (NAFLD) has become the most common form of chronic liver disease in both children and adults, affecting up to 30% of the American population [1], [2]. NAFLD encompasses a wide spectrum of conditions associated with an over-accumulation of fat in the liver, ranging from hepatic steatosis to steatohepatitis (NASH) and cirrhosis [3]. Hepatic steatosis is characterized by an isolated accumulation of lipids in the liver and is generally thought to follow a relatively benign, non-progressive clinical course [4]. NASH, on the other hand, is a serious condition with about 5 to 25% of patients progressing to fibrosis and cirrhosis with the associated complications of portal hypertension, liver failure and hepatocellular carcinoma [3], [5]. Liver biopsy, an invasive procedure associated with possible significant complications, presently remains the only reliable method to differentiate hepatic steatosis from NASH [6], [7] and monitor any response to therapeutic interventions. Therefore, there is currently a significant interest in in developing non-invasive tests for this condition [8].

Extracellular vesicles (EVs) are small membrane vehicles released from dying or activated cells. There are two main populations of EVs, namely exosomes (EXO) and microparticles (MPs), which differ in size, composition and mechanism of generation. Exosomes are small, 30–100 nm in diameter, and are released by exocytosis as a result of multivesicular bodies fusing with the plasma membrane. MPs are small structures surrounded by a phospholipid bilayer and have a diameter between 100–1000 nm. MPs are generated and released through a controlled budding/blebbing of the plasma membrane. This process involves a regulated sorting of bioactive molecules into the shed MP. Moreover, MPs feature a flipping of phosphatidylserine from the inner to the outer membrane during cellular activation or early apoptosis and they can be isolated by differential ultracentrifugation and FACS analyses [9]. EVs are key cell-to-cell communicators because they carry signatures from parenteral cells such as surface receptors, proteins (membrane, cytosolic and nuclear), RNAs (including mRNAs and microRNAs) and lipids [10]–[12]. EVs deliver these molecular packets of information to other cells through an interaction with surface receptors or internalization [13], [14]. Notably, released EVs do not only stay in the tissue of origin, but also circulate in the blood stream. Indeed, recent studies from our group and others have demonstrated that primary and immortalized hepatocytes are capable of producing and releasing the two main subtypes of EVs: exosomes and MPs [15]–[18]. Moreover, we further demonstrated that EVs are formed and released during the accumulation of lipotoxic lipids in hepatocytes, which is a key mechanism of liver damage and disease progression in NAFLD [18], [19]. In vivo studies in bile duct-ligated rats have found increased circulating MPs, while two recent pilot studies in humans showed increased levels of inflammatory cell-derived MPs in patients with NAFLD and in patients with alcohol and/or chronic hepatitis C related cirrhosis [15], [20], [21]. Thus, in this study we examined whether EVs are increased in liver and blood during experimental NAFLD. In order to better understand whether EVs may be novel non-invasive biomarkers to monitor liver damage in NAFLD we aimed to assess a detailed characterization of EV populations released during experimental NAFLD development and the utility of monitoring and quantifying EVs in blood as biomarkers of liver damage.

## Materials and Methods

### Animal studies

Male C57BL/6 wild type mice, 20 to 25 gm of body weight, 7 weeks old, were placed on a Choline Deficient L-Amino Acid (CDAA) (Dyets, Bethlehem, PA, USA) diet or on one of two control diets (Choline Supplemented L-Amino Acid, (CSAA); and normal chow) for 4, 8 or 20 weeks. This diet (CDAA) has been extensively shown to result in steatosis associated with significant inflammation and progressive fibrosis, which is pathologically similar to human severe steatohepatitis [22]. Additionally n = 6 mice were placed on a high fat diet (HF) containing 45% kcal from fat, 18.8 kJ/g (Research Diets, New Brunswick, NJ, USA) or normal chow diet for 12 weeks to investigate our hypothesis on a different model of experimental NAFLD [23]. Mice were sacrificed and the liver and blood were collected under anesthesia achieved by i.p. injecting with a 21G needle, a mixture of 100 mg/Kg of Ketamine and 10 mg/Kg of Xylazine dissolved in a 0.9% saline solution [24]. The studies were approved by the University of California San Diego Institutional Animal Care and Use Committee and followed the National Institutes of Health guidelines outlined in “Guide for the Care and Use of Laboratory Animals”.

### Histopathology, immunohistochemistry and plasma assays

Plasma alanine aminotransferase (ALT) activity was measured by using a commercial kit (Sekisui Diagnostics, LLC, Framingham, New York). Liver tissue was fixed in 10% formalin. Specimens were routinely processed, embedded in paraffin, sectioned at 10 µm and stained with hematoxylin and eosin (H&E). Hepatic steatosis, inflammation and ballooning were assessed in NAFLD/NASH and control mice by an experienced pathologist (Dr. Bettina G. Papouchado) in a blinded fashion. Scoring analysis was based on NAFLD activity score (NAS) [25]. Immunohistochemistry for detection of pathological angiogenesis was performed using an anti-CD-31polyclonal rabbit antibody (1∶25; Abcam, Cambridge, MA) as previously described [18]. For detection of tissue collagen deposition, a Sirius red (saturated picric acid containing 0.1% Direct Red 80 and 0.1% Fast Green FCF) staining was performed. To detect cell death in paraffin-embedded liver specimens the ApopTag peroxidase in situ apoptosis detection kit (Millipore, Billerica, MA, USA) was used according to manufacturer’s instructions. Sirius red positive areas and TUNEL-positive cells were analyzed in five random low-power views on each slide using Image J Software (NIH). 10, 20 or 40X magnifications were used for imaging.

### Isolation of circulating extracellular vesicles

Circulating extracellular vesicles were isolated from fresh blood samples harvested from CDAA, CSAA, HF and normal chow fed mice. Approximately 1 mL of whole blood was collected in heparin-conditioned 1.5 mL tubes and centrifuged at 1,200 g for 15 minutes in order to obtain platelet-poor plasma (PPP). Supernatant containing EVs was centrifuged at 12,000 g for 12 minutes in order to get the platelet-free plasma (PFP), as described previously [26]. To differentially isolate microparticles and exosomes, PFP was additionally ultracentrifuged at 20,000 g for 30 minutes at 10°C (SW41, Beckman, Indianapolis, IN, USA) to pellet microparticles. The MP-free supernatant was transferred to a new tube and ultracentrifuged at 100,000 g for 1 h at 10°C to pellet exosomes. The size of MPs and exosomes was determined by a Dynamic Light Scattering Zetasizer (Malvern, Worcestershire, UK).

### Flow cytometry

Circulating extracellular vesicle acquisition was performed by means of the BD LSRII Flow Cytometer System (BD Biosciences, San Jose, CA, USA) and the data were analyzed using FlowJo software (TreeStar Inc., Ashland, OR, USA). A volume of 30 µL of PFP was filtered on a 0.22-µm filter (Millipore) and incubated with 1 µM of Calcein AM (BD Biosciences, San Jose, CA, USA) in PBS for 1 h at 37°C. Calcein AM, a non-fluorescent marker that becomes fluorescent (emission wavelength of 515 nm) upon cleavage by cytosolic esterases, stains only intact extracellular vesicles and it is not reactive with cellular fragments. Standardization of the protocol was achieved using 1 µm latex fluorescent beads (Sigma-Aldrich, St Louis, MO, USA) and ultraviolet 2.5 µm flow cytometry alignment beads (Invitrogen, Grand Island, NY, USA). Forward (FS) and side scatter (SS) parameters were plotted on logarithmic scales to best cover a wide size range. Single staining controls were used to check fluorescence compensation settings and to set up positive regions. Lightning-link conjugation kit (Novus Biologicals, Littleton, CO, USA) was used to generate phycoerythrin (PE)-conjugated anti-mouse liver carboxylesterase monoclonal antibody (Abcam). Circulating EVs isolated from mice fed with CSAA and CDAA diet for 20 weeks were double-labelled with Calcein AM and liver carboxylesterase to quantify liver-derived EVs in the whole vesicle population.

### Sucrose gradient

For the proteomics analysis, platelet-free plasma samples were purified on a 10–70% sucrose gradient as previously described [27], with some modifications. Approximately 2 mL of PFP isolated from CDAA- and CSAA-fed mice for 20 weeks were layered on top of the sucrose gradient in SW41 tubes (Beckman Coulter Inc., Brea, CA, USA) and ultracentrifuged at 100,000 g for 18 h at 10°C. Fractions comprised between 1.19 and 1.26 g/mL^−1 ^were collected, resuspended in PBS and further ultracentrifuged at 100,000 rpm for 1 h at 10°C to wash out sucrose from EV preparation. For the EV characterization, the same fractions were ultracentrifuged at 20,000 g for 30 min at 10°C to pellet the microparticles. The supernatant was transferred to a new tube and centrifuged at 100,000 g for 1 h at 10°C to pellet exosomes. The resulting purified microparticles and exosomes were resuspended in PBS and processed for western blot, FACS and dynamic light scattering analyses.

### Sample preparation for MS

Protein samples were diluted in TNE (50 mM Tris pH 8.0, 100 mM NaCl, 1 mM EDTA) buffer. RapiGest SF reagent (Waters Corp.) was added to the mix to a final concentration of 0.1% and samples were boiled for 5 min. TCEP (Tris (2-carboxyethyl) phosphine) was added to 1 mM (final concentration) and the samples were incubated at 37°C for 30 min. Subsequently, the samples were carboxymethylated with 0.5 mg/ml of iodoacetamide for 30 min at 37°C followed by neutralization with 2 mM TCEP (final concentration). Protein samples prepared as above were digested with trypsin (trypsin:protein ratio –1∶50) overnight at 37°C. RapiGest was degraded and removed by treating the samples with 250 mM HCl at 37°C for 1 h followed by centrifugation at 14000 rpm for 30 min at 4°C. The soluble fraction was then added to a new tube and the peptides were extracted and desalted using Aspire RP30 desalting columns (Thermo Scientific).

### LC-MS-MS analysis

Trypsin-digested peptides were analyzed by high pressure liquid chromatography (HPLC) coupled with tandem mass spectroscopy (LC-MS/MS) [28]. The nanospray ionization experiments were performed using a TripleTof 5600 hybrid mass spectrometer (ABSCIEX) interfaced with nano-scale reverse-phase HPLC (Tempo) using a 10 cm-100 micron ID glass capillary packed with 5-µm C18 ZorbaxTM beads (Agilent Technologies, Santa Clara, CA). Peptides were eluted from the C18 column into the mass spectrometer using a linear gradient (5–60%) of ACN (Acetonitrile) at a flow rate of 250 µl/min for 1 h. The buffers used to create the ACN gradient were: Buffer A (98% H2O, 2% ACN, 0.2% formic acid, and 0.005% TFA) and Buffer B (100% ACN, 0.2% formic acid, and 0.005% TFA). MS/MS data were acquired in a data-dependent manner in which the MS1 data were acquired for 250 ms at m/z of 400 to 1250 Da and the MS/MS data were acquired from m/z of 50 to 2,000 Da. For Independent data acquisition (IDA) parameters MS1-TOF 250 milliseconds, followed by 50 MS2 events of 25 milliseconds each. The IDA criteria, over 200 counts threshold, charge state +2–4 with 4 seconds exclusion. Finally, the collected data were analyzed using MASCOT (Matrix Sciences) and Protein Pilot 4.0 (ABSCIEX) for peptide identifications [29], [30]. The SEQUEST algorithm was used to match MS/MS spectra to peptides in the sequence databases [31], [32]. Data of EV proteins isolated from CDAA and CSAA-fed mice were normalized to those obtained from normal chow-fed mice.

### Electron microscopy

For transmission electron microscopy, extracellular vesicles were adhered to 100 mesh Formvar and carbon coated grids for 5 minutes at room temperature. Grids were washed once with water, stained with 1% uranyl acetate (Ladd Research Industries, Williston VT) for 1 minute, dried and viewed using a JEOL 1200 EXII transmission electron microscope. Images were captured using a Gatan Orius 600 digital camera (Gatan, Pleasanton, CA). Liver samples were collected from the CDAA-fed mice after a short liver perfusion with 10 mL of 4% paraformaldehyde in 0.15 M sodium cacodylate buffer, pH 7.4 by using a 21 G needle. Samples were immersed in modified Karnovsky’s fixative (2.5% glutaraldehyde and 2% paraformaldehyde in 0.15 M sodium cacodylate buffer, pH 7.4) for at least 4 hours, postfixed in 1% osmium tetroxide in 0.15 M cacodylate buffer for 1 hour and stained en bloc in 3% uranyl acetate for 1 hour. Samples were dehydrated in ethanol, embedded in Durcupan epoxy resin (Sigma-Aldrich), sectioned at 50 to 60 nm on a Leica UCT ultramicrotome, and picked up on Formvar and carbon-coated copper grids. Sections were stained with 3% uranyl acetate for 5 minutes and Sato’s lead stain for 1 minute. Grids were viewed using a JEOL 1200EX II (JEOL, Peabody, MA) transmission electron microscope and photographed using a Gatan digital camera (Gatan, Pleasanton, CA).

### Immunoprecipitation of miR-122 associated with Argonaute-2 (Ago2) complexes

Platelet-free plasma isolated from CDAA and CSAA-fed mice for 20 weeks was centrifuged for 1 h at 30,000 rpm at 10°C to pellet extracellular vesicles (EV). A volume of 200 µL of vesicles-free supernatant was diluted in 200 µL (1∶1) of PBS solution and immunoprecipitated as detailed previously [33]. Amount of miR-122 encapsulated in EVs and associated with Ago2 was determined by miRNeasy Mini kit (QIAGEN). CDNA was synthesized using specific miRNA primers (Applied Biosystems) in TaqMan microRNA Reverse Transcription kit (Applied Biosystems). MiRNA abundance was detected with TaqMan probe (Applied Biosystems) on 7300 Real time PCR system (Applied Biosystems). The U6 snRNA was used as an internal control.

### Real-time PCR and miRNA expression

Liver samples were harvested from mice and homogenized. Total RNA was isolated using RNeasy kit (Qiagen, Valencia, CA) and reverse transcribed by iScript cDNA synthesis kit (Bio-Rad) according to the manufacturer’s instructions. Quantitative Real time PCR was performed on a BioRad Cycler (Biorad, Hercules, CA, USA) by using SYBRGreen real time PCR master mix (Kapabiosystem, Woburn, MA, USA) according to the manufacturer’s instructions. The housekeeping gene 18S was used as an internal control. The PCR primers used to amplify each gene are listed in Table S2. For isolation and quantification of miR-122 and miR-192, platelet-free plasma was isolated from CDAA-fed, high fat or control mice and incubated with 10 µg/mL of RNase (Roche, Indianapolis, IN, USA) for 30 min at 37°C to remove any RNAs adhering to the external leaflet of circulating EVs. Circulating EVs were then ultracentrifuged at 100,000 g for 90 min at 10°C. Total encapsulated RNAs in EVs or in liver specimens, including miRNAs, were isolated by miRNeasy Mini kit (QIAGEN). CDNA was synthesized using specific miRNA primers (Applied Biosystems) in TaqMan microRNA Reverse Transcription kit (Applied Biosystems). MiRNA abundance was detected with TaqMan probe (Applied Biosystems) on 7300 Real time PCR system (Applied Biosystems). The U6 snRNA was used as an internal control and to normalize miR-122 and miR-192 expression.

### Western blot analysis

Extracellular vesicles were isolated from blood samples as described in the ‘MPs isolation and purification’ paragraph. Approximatively 10 µg of EV protein lysates were solubilized in Laemli buffer, resolved by a 4–20% Criterion Tris-HCl gel electrophoresis system(Biorad, Hercules, CA, USA) and transferred to a 0.2 µm nitrocellulose membrane (Biorad, Hercules, CA, USA). Primary rabbit polyclonal antibody anti-mouse Vanin-1 (1∶500; Proteintech, Chicago, IL, USA), Cd63, Cd81, ASGPR1 (1∶1000; Genetex, Irvine, CA, USA) and Icam-1 (1∶1000; Abnova, Taipei city, Taiwan) were incubated overnight at 4°C. Proteins were visualized by Supersignal West Pico chemiluminescence substrate (Pierce biotechnology, Rockford, IL, USA) and quantified by ImageJ software.

### Statistical analysis

All data were expressed as the mean ± SD unless otherwise indicated. Differences between three or more groups were compared by a nonparametric Kruskal-Wallis ANOVA test. If a significant effect was detected, post-hoc pair-wise comparisons were performed using Mann-Whitney tests with Bonferroni correction. Differences between two groups were compared by a two-sided Student’s t-test if the data were normal or a Mann-Whitney test if the data deviated from the normal distribution. Differences were considered to be statistically significant at P<0.05. Spearman’s correlation coefficient was calculated for the correlation analyses and an r^2^ value higher than 0.7 was considered a strong correlation. All statistical analyses were performed using GraphPad Prism 4.0c (La Jolla, CA, USA) or R v3.0.2 (www.r-project.org).

## Results

### Diet-induced NAFLD promotes changes in the levels of EVs that are early markers of disease severity and reflect changes in liver histopathology

**Figure 1:**
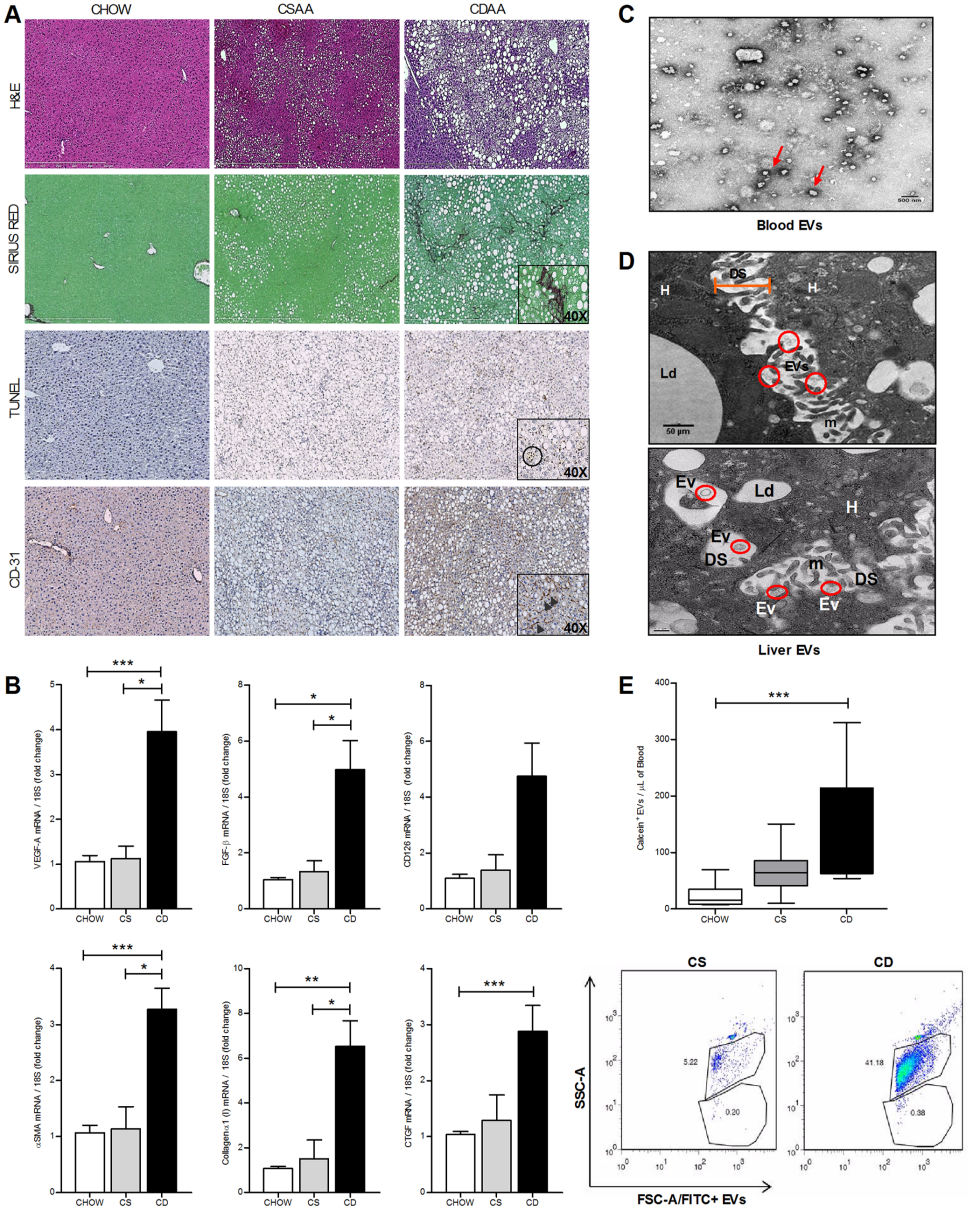
CDAA resembles the histopathological features of human NASH. (**A**) Liver specimens from wild type C57BL/6 mice (n = 12) fed with the CDAA (CD), CSAA (CS) or chow diet for 20 weeks, were used for assessing liver damage (Hematoxylin & eosin staining, H&E), fibrosis by detecting the collagen deposition (Sirius red staining), cell death (TUNEL staining) and pathological angiogenesis (CD31 staining). (**B**) Analysis of the expression of the transcripts for VEGF-A, FGF-β, CD126 (VE-cadherin), Collagen type-I, α-smooth muscle actin (α-SMA) and CTGF, assessed by quantitative real-time PCR. The housekeeping gene 18S was used as internal control. (**C**) Representative TEM microphotograph of circulating EVs isolated from CDAA-fed mice for 20 weeks. (**D**) Representative microphotograph of hepatic extracellular vesicles (EV, red circle) in the space of Disse (DS). Hepatocyte (H), (Ld) lipid droplet, (m) microvilli. (**E**) Flow cytometry analysis (Whisker plot and dot plot) of circulating Calcein+ extracellular vesicles per µL of platelet-free plasma isolated from CDAA (CD, n = 12), CSAA (CS, n = 12) or chow diet fed mice (n = 12) for 20 weeks. Values represent mean ± SD. *P<0.05, **P<0.005, ***P<0.0005, Kruskal-Wallis test with post-hoc Mann-Whitney test and Bonferroni correction.

**Figure 2:**
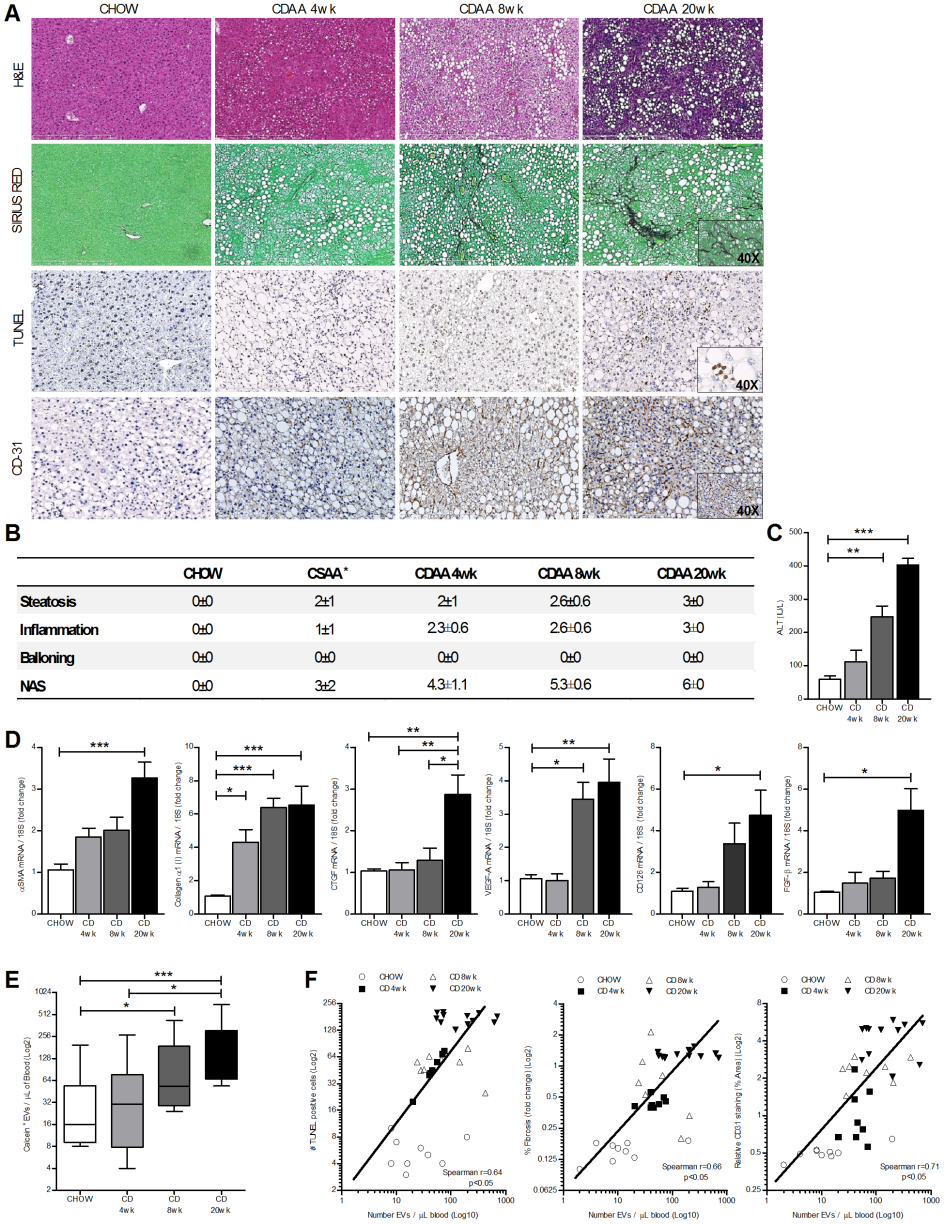
Release of circulating extracellular vesicles is time-dependent and correlates with the histopathological features of NASH. C57BL/6 mice (n = 8–12) were fed with CDAA (CD) or chow diet for 4 weeks, 8 weeks or 20 weeks to mimic an early stage, mild stage or severe stage of NAFLD, respectively. (**A**) Representative microphotographs of hematoxylin & eosin staining, collagen fibers deposition (Sirius red staining), cell death (Tunel staining) and endothelial cells (CD31 immunostaining) in liver specimens harvested from mice fed a chow or CDAA diet for 4, 8 or 20 weeks. (**B**) H&E liver specimens were analyzed in a blinded way for steatosis, inflammation and ballooning and NAS score was determined in mice fed with CDAA diet for 4, 8 or 20 wks or with control diets (chow or CSAA, *data shown in Fig. 1). (**C**) Plasma alanine aminotransferase (ALT). (**D**) qPCR of the pro-fibrogenic markers α-SMA, Collagen-Iα1 and connective tissue growth factor (CTGF) and pro-angiogenic markers VEGF-A, FGF-β and VE-Cadherin (CD126) in chow or CDAA fed mice for 4, 8 or 20 weeks. Mean values were normalized to 18S housekeeping gene. (**E**) Whisker plot of flow cytometry analysis of Calcein-FITC-positive extracellular vesicles isolated from platelet-free plasma of chow or CDAA fed mice for 4, 8 or 20 weeks. (**F**) Spearman correlations between circulating level of extracellular vesicles and cell death (TUNEL positive cells), hepatic fibrosis or endothelial cells (CD31-positive cells) in mice fed with chow or CDAA diet for 4, 8 or 20 weeks. *P<0.05, **P<0.005, ***P<0.0005, Kruskal-Wallis test with post-hoc Mann-Whitney test and Bonferroni correction.

To investigate whether EVs are produced and released in NAFLD, we placed C57BL/6 mice on a Choline Deficient L-Amino Acid (CDAA) or one of two control diets (CSAA or normal chow). We observed that mice fed with the CDAA diet developed significant macro-vesicular steatosis, marked inflammation, cell death, pathological angiogenesis and fibrosis (Fig. 1A–B). By employing electron microscopy we were able to identify a large presence of EVs in both liver specimens and blood of NAFLD mice (Fig. 1C–D). Importantly, we observed that EVs were predominantly detected between the hepatocyte villi and the sinusoid wall (Fig. 1D). Additionally, FACS analysis identified a marked increase of Calcein-positive EVs in blood isolated from CDAA fed mice compared to CSAA and chow fed mice (Fig. 1E). Our findings of increased levels of circulating EVs in mice with established severe NAFLD led us to further examine the potential role of these vesicles as noninvasive monitoring tools of disease progression over time and pathohistological severity. In order to address these questions, male C57BL/6 mice were placed on the CDAA, CSAA or a regular chow diet for 4, 8 or 20 weeks. These time points have been shown to be associated with early, mild and established NAFLD, respectively [22]. We observed that the increase in circulating EVs isolated from platelet-free plasma was time-dependent. Determination of liver fibrosis, cell death and pathological angiogenesis were further assessed respectively by Sirius red staining for collagen deposition, TUNEL staining and immunostaining for CD-31, a marker of endothelial cells. Mice placed on the CDAA diet for 4 weeks showed early outcomes of liver injury, mice on the CDAA diet for 8 weeks developed mild liver fibrosis, angiogenesis and cell death, while mice on the CDAA diet for 20 weeks developed severe cell death, hepatic fibrosis and pathological angiogenesis (Fig. 2A). These findings were also confirmed by the histopathological analysis of NAFLD Activity Score (Fig. 2B), level of plasma alanine aminotransferase (ALT) (Fig. 2C) and by the analysis of the transcripts for pro-fibrogenic (α-Smooth Muscle Actin, CollagenIα1, Connective Tissue Growth Factor) and pro-angiogenic genes (Vascular Endothelial Growth Factor-A, CD126, Fibroblast Growth Factor-β) (Fig. 2D), which indicated a time-dependent progression of all the main features of liver injury associated with NAFLD. FACS analysis showed that the number of circulating EVs increased over time and became significantly higher by 8 weeks on the CDAA diet, a time point associated with progressive changes from mild NAFLD (Fig. 2E). Indeed, Spearman correlation analyses showed a significant correlation with EV level and ratio of dead cells (r^2^ = 0.64, p<0.05), extent of liver fibrosis (r^2^ = 0.66, p<0.05) and pathological angiogenesis (r^2^ = 0.71, p<0.05) (Fig. 2F). These findings suggest that blood EVs are released by damaged or stressed hepatic cells during NAFLD development in a time-dependent manner and their levels strongly correlate with the extent of liver damage.

### Circulating EVs in blood from NAFLD animals are predominantly MPs and have a distinct antigenic composition

**Figure 3:**
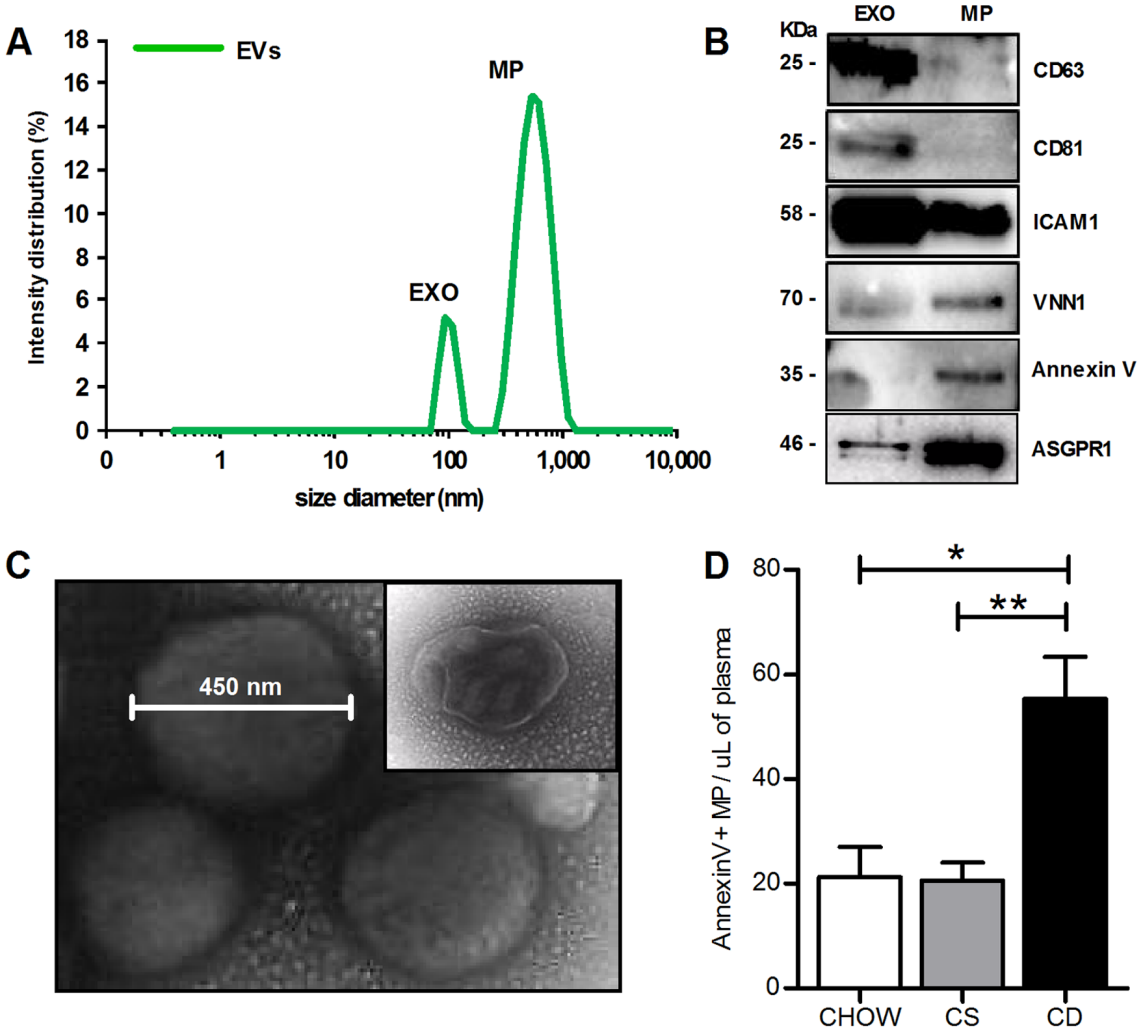
Characterization of circulating extracellular vesicles (EV) in a diet-induced NASH model. (**A**) Dynamic light scattering analysis was performed to determine the size of EVs isolated from the platelet-free plasma of CDAA-fed mice for 20 weeks. The graph shows a predominant peak of big vesicles (mean 630 nm) corresponding to microparticles and a peak of smaller vesicles (mean 100 nm), corresponding to exosomes. (**B**) Exosomes (EXO) and microparticles (MP) were separated from the whole EV population and markers of EXO (Cd63, Cd81, ICAM-1), MP (Vanin-1 and Annexin V) and the hepatocyte-specific asialoglycoprotein-receptor (ASGPR1) were identified by western blot analysis. (**C**) Representative TEM macro photograph of circulating EVs isolated from platelet-free plasma of CDAA-fed mice for 20 weeks. (**D**) Flow cytometry analysis of circulating AnnexinV+ microparticles per µL of platelet-free plasma isolated from CDAA (CD), CSAA (CS) or chow diet fed mice for 20 weeks. *P<0.05, **P<0.005, ***P<0.0005, Kruskal-Wallis test with post-hoc Mann-Whitney test and Bonferroni correction.

The findings of increased blood EVs in NAFLD as early markers of liver injury in this condition led us to further explore the characteristics of these EVs. We next performed a series of studies, including dynamic light scattering analysis and FACS analysis, which identified two populations of extracellular vesicles released in circulation during NAFLD development: microparticles (MP) and exosomes (EXO) (Fig. 3). Indeed, analysis of EV size identified two distinct peaks, a large predominant peak that corresponded to EVs with a diameter between 100 and 1,000 nm (mean 630 nm) consistent with the size previously reported for MPs [21], and a second small peak of EVs with a diameter between 30 and 100 nm (mean 100 nm) consistent with the size previously reported for exosomes [34], [35] (Fig. 3A). In order to confirm the presence of EXO and MP in the whole population of purified circulating extracellular vesicles isolated from the CDAA-fed mice for 20 weeks, we performed western blot analyses for some exosomal markers (Cd63, Cd81, Icam1), MP markers (AnnexinV and Vanin-1 (VNN1) - the latter of which has recently been identified as a novel surface ectoenzyme present in MPs released by stressed hepatocytes [18]), and for the hepatocyte-specific asialoglycoprotein-receptor (ASGPR1), which was recently reported in MPs released during post-ischemic reperfusion [36] (Fig. 3B). The presence of circulating MPs was further confirmed by transmission electron microscopy (Fig. 3C) and flow cytometry analysis of AnnexinV positive MPs in plasma of NAFLD mice (Fig. 3D).

To determine whether the EV population present in blood from NAFLD animals could be profiled based on the antigenic composition, we next focused on the characterization of the antigenic composition of blood EVs in NASH. Indeed, in order to identify potential protein targets present in blood EVs during NASH development, we performed a proteomic analysis of purified blood EVs. For these studies we obtained EVs from platelet-free plasma collected from mice fed the CDAA or one of two control diets (CSAA and normal chow) for 20 weeks. We then purified the EVs using sucrose gradient ultracentrifugation (Fig. S1) and, lastly, we ascertained the EV proteome by LC-MS/MS analysis. Importantly, the proteome of blood EVs isolated from CDAA mice differed from that of control mice (Table S1). Table 1 lists all unique proteins identified in three independent preparations of blood extracellular vesicles isolated from CDAA-fed mice.

**Table 1:** Protein identified by LC-MS/MS in circulating extracellular vesicles isolated from CDAA-induced NASH mice.

	Gene symbol	Gene ID	Protein Description	Peptides (95%) ^a^	% Cov ^b^	SpC ^c^
**Cytoskeletal remodelling/vesiculation**	KRT6A	16687	Keratin, 6A, type II	20	47.87	47
	ACT	11459	Actin	6	23.47	34
	ACTG1	11465	Actin, gamma, 1	6	23.47	19
	ADD2	11519	Beta-adducin	1	4.69	1
	JUP	16480	Desmoplakin	1	0.31	1
	PLAK	16480	Junction plakoglobin	4	8.32	6
	KRT5	110308	Keratin 5	22	34.48	46
	DMTN	13829	Dematin	1	3.13	2
	KRT7	110310	Keratin 7, type II	2	10.72	21
	VCP	269523	Transitional endoplasmic reticulum ATPase	3	6.7	3
	SPTB	20742	Spectrin beta 1	27	21.08	46
	SPTA1	20739	Spectrin alpha chain	32	24.8	65
	KRT77	406220	Keratin 77	4	18.18	24
	MPP1	17524	Membrane protein, palmitoylated	2	12.61	3
	KRT17	16667	Keratin 17, type I	10	39.26	26
	KRT25	70810	Keratin 25, type I	1	12.44	11
	EPB41	269587	Erythrocyte protein band 4.1	6	12.89	8
	GYPA	14934	Glycophorin A	1	18.45	1
	LRG1	76905	Leucine-rich HEV glycoprotein	1	4.09	1
	KRT1	16678	Keratin 1, type II	5	13.97	63
	KRT2	16681	Keratin 2, type II	9	22.49	24
	KRT73	223915	Keratin 73, type II	6	22.08	21
	KRT10	16661	Keratin 10	7	12.11	100
	NEST	18008	Nestin	1	2	2
	KRT6B	16688	Keratin 6B, type II	5	6.01	26
	KRT42	68239	Keratin 42, type I	4	8	8
**Signalling/Chaperone/Transcription regulation**	PRDX2	21672	Peroxiredoxin-2	2	9.59	3
	ZNF521	22696	Zinc finger protein 521 isoform 1	1	2.74	2
	REFBP2	56009	RNA and export factor-binding protein 2	1	15.14	2
	APOA1	11806	Apolipoprotein A–I	10	37.88	30
	VWF	22371	von Willebrand factor	2	0.81	2
	AQP1	11826	Aquaporin-1	2	10.78	7
	ECM1	13601	Extracellular matrix protein 1	1	10.55	3
	STOML3	13830	Stomatin	1	4.06	1
	BACH1	12013	BTB and CNC homology 1	1	5.82	1
	ATM	11920	Serine-protein kinase ATM	2	4.69	2
	GABRA5	110886	Gamma-aminobutyric acid receptor subunit alpha-5	1	3.35	1
	LGALS3BP	16854	Galectin-3-binding protein	1	4.67	2
	MVP	78388	Major vault protein	1	1.04	1
	TSKU	244152	Tsukushi	1	9.605	1
	TF	22041	Serotransferrin	48	58.82	323
	PRDX2	21672	Peroxiredoxin-2	6	31.82	14
	HMGA1	15361	High mobility group AT-hook 1	1	23.36	1
	UBC	22190	Ubc protein	1	19.24	3
	CHRNA10	504186	Cholinergic receptor, nicotinic, alpha polypeptide 10	1	2.01	1
	ASAP2	211914	Arf-GAP with SH3, PH domains, ANK repeat 2	1	0.82	3
	PRDX1	18477	Peroxiredoxin-1	2	14.06	4
	PRDX4	53381	Peroxiredoxin-4	1	3.49	2
**Enzymes/Metabolic processes**	FABP5	16592	Fatty acid binding protein 5	1	6.66	1
	ADIPOQ	11450	Adiponectin	2	8.9	4
	ALDOA	11674	Fructose-bisphosphate aldolase	1	6.31	1
	APOA2	11807	Apolipoprotein A-II	8	71.57	25
	HBB-A1	15122	Alpha-globin 1	8	39.44	300
	ORM1	18406	Alpha-1-acid glycoprotein 1	1	4.34	7
	SERPIN-1A	20700	Alpha-1-antitrypsin 1–1 (Serpin 1A)	6	13.56	21
	SERPIN-1B	20701	Alpha-1-antitrypsin 1–2 (Serpin 1B)	6	13.56	21
	SERPIN-1C	20702	Alpha-1-antitrypsin 1–3 (Serpin 1C)	6	13.35	22
	SERPIN-1D	20703	Alpha-1-antitrypsin 1–4 (Serpin 1D)	5	11.38	20
	SERPIN-1G	18787	Serine protease inhibitor G1	5	26.19	28
	SERPIN-3A	20714	Serine protease inhibitor A3A	2	15.55	3
	SERPIN-2F	18816	Alpha-1-antitrypsin-related protein	2	8.14	5
	APOA4	11808	Apolipoprotein A-IV	1	4.55	1
	APOE	11816	Apolipoprotein E	1	7.39	1
	EPB42	13828	Erythrocyte protein band 4.2	6	12.47	10
	FTL1	14319	Ferritin	1	16.39	1
	GLUL	14645	Glutamine synthetase	4	14.48	6
	GPX3	14778	Glutathione peroxidase 3	3	22.12	3
	HP	15439	Haptoglobin	13	46.11	45
	ALDH1A1	11668	Aldehyde dehydrogenase family 1	3	21.36	12
	GAPDH	14433	Glyceraldehyde-3-phosphate dehydrogenase	2	15.62	3
	HSPA1L	15511	Heat shock 70 kDa protein 1	1	7.95	1
	HPX	15458	Hemopexin	16	49.78	52
	PON1	18979	Serum paraoxonase/arylesterase 1	12	33.21	46
	COLEC11	71693	Collectin-11 isoform II	1	8.27	1
	PZP	11287	Pregnancy zone protein	153	60.2	253
	MGAM	232714	Maltase-glucoamylase	2	3.83	2
	ALDH1L1	107747	Cytosolic 10-formyltetrahydrofolate dehydrogenase	2	14.26	4
	MUG1	17836	Murinoglobulin-1	8	8.4	23
	NUB1	53312	Nedd8 ultimate buster-1	1	2.93	1
	PSMA1	19165	Proteasome subunit alpha type 1	2	14.83	3
	PSMA3	19167	Proteasome subunit alpha type 3	2	17.09	9
	PSMA4	26441	Proteasome subunit alpha type 4	1	10.34	5
	PSMA6	19171	Proteasome subunit beta type	4	23.58	10
	PSMB1	19170	Proteasome subunit beta type 1	4	37.08	14
	PSMB6	19175	Proteasome subunit beta type 6	4	25.74	8
	PSMB7	19177	Proteasome subunit beta type 7	1	20.58	4
	HGD	15233	Homogentisate 1, 2-dioxygenase	3	21.8	4
	LAP3	66988	Leucine aminopeptidase 3	2	30.83	3
	RBP4	19662	Retinol binding protein 4, plasma	1	4.97	3
	GC	14473	Vitamin D-binding protein	1	1.89	1
	APCS	20219	Serum amyloid P-component	1	14.73	2
	DNPEP	13437	Aspartyl aminopeptidase	3	7.02	5
	DUSP9	75590	Dual specificity protein phosphatase	1	7.74	1
	CANT1	76025	Calcium activated nucleotidase 1	1	3.18	2
	CPB2	56373	Carboxypeptidase B2	2	3.79	1
	CPN1	93721	Carboxypeptidase N, polypeptide 1	3	14.88	9
	CPN2	71756	Carboxypeptidase N, polypeptide 2	2	12.98	9
	CPB2	56373	Carboxypeptidase B2	2	3.79	1
	GGTA1	14594	N-acetyllactosaminide alpha-1,3-galactosyltransferase	3	38.62	23
	GPLD1	14756	Glycosylphosphatidylinositol specific phospholipase D1	4	11.4	11
	H6PD	14381	Hexose-6-phosphate dehydrogenase	1	2	1
	UBE4B	63958	Ubiquitination factor E4B	1	1.87	2
	ALB	11657	Albumin	114	70.07	7986
	SLC4a3	20533	Band 3 anion transport protein	8	13.78	57
	SERPIN-K3	20714	Serine protease inhibitor A3K	3	13.64	3
	TTR	22139	Transthyretin	1	19.05	4
	CP	12870	Ceruloplasmin	11	15.75	25
	CES1c	13884	Liver carboxylesterase 1	4	17.51	10
	OXR1	170719	Oxidation resistance protein 1	1	1.27	2
**Fibrinolysis/Coagulation**	A2M	232345	Alpha-2-Macroglobulin	984	81.15	12620
	KNG1	16644	Kininogen-1	2	4.37	4
	KLK1	16612	Kallikrein B	4	21.94	11
	F2	14061	Coagulation factor II	2	6.63	5
	F13a1	74145	Coagulation factor XIII, A1 subunit	1	14.07	1
	SERPIN-C1	11905	Antithrombin (Serpin C1)	2	11.4	5
	PLAU	18787	Plasminogen	4	11.82	11
	FGB	110135	Fibrinogen, B beta polypeptide	9	24.95	18
	FGA	14161	Fibrinogen, A alpha polypeptide	5	16.7	14
	FGG	14188	Fibrinogen, gamma polypeptide	34	51.38	134
	SERPIN-G1	12258	Plasma protease C1 inhibitor (Serpin-G1)	5	16.43	24
**Complement system/Immunity**	AHSG	11625	Alpha-2-HS-glycoprotein	2	24.06	4
	ANK1	17733	Ankyrin 1	18	20.61	36
	C1rA	50909	Complement C1r, subcomponent A	16	34.51	62
	C1qA	12259	Complement C1q, subcomponent subunit A	8	40.41	40
	C1qB	12260	Complement C1q subcomponent subunit B	10	25.69	64
	C1qC	12262	Complement C1q subcomponent subunit C	7	30.49	56
	C1qbp	12261	Complement C1q subcomponent binding protein	9	29.65	45
	C1s	50908	Complement C1s subcomponent A	22	37.75	84
	C3	12266	Complement C3	37	33.61	102
	C4	12268	Complement C4	27	22.6	13
	C5	15139	Complement C5	16	21.49	49
	C8γ	69379	Complement C8 polypeptide gamma	2	28.71	5
	C9	12279	Complement C9	1	3.38	1
	CD47	16423	Leukocyte surface antigen CD47	1	7.59	1
	IgJ	16033	Immunoglobulin J chain	10	44.65	80
	Igk	14972	Immunoglobulin k chain	1	10.32	7
	Igγ1	111507	Immunoglobulin γ chain 1	2	30.26	9
	Igγ2b	111507	Immunoglobulin γ chain 2b	2	10.21	11
	FCN1	14133	Ficolin-1	1	2.69	2
	MASP2	17174	Mannan-binding lectin serine protease 1	2	6.95	4
	MBL1	17194	Mannose-binding lectin-1	3	15.16	13
	PIGR	18703	Polymeric immunoglobulin receptor	7	14.4	13
	CAMP	12796	Cathelin-related antimicrobial peptide	2	23.91	4
	CRP	12944	C-reactive protein	8	33.78	24
**Apoptosis**	CTSD	13033	Cathepsin-D	1	7.86	1
	GSN	227753	Gelsolin	4	26.41	7
	CRYAB	12955	Crystallin, alpha B	4	27.43	8
	CLU	12759	Clusterin	3	14.73	7
	CD5L	11801	CD5 antigen-like	16	41.76	81
**Angiogenesis/Cell adhesion/migration**	RETNLG	57262	Myeloid cysteine-rich protein	7	34.19	21
	IAP	16423	Leukocyte surface antigen CD47	1	7.59	1
	DSG1A	13510	Desmoglein 1 alpha	1	1.22	4
	SHC1	20416	SHC-transforming protein 1	1	2.85	1
	DSG1B	225256	Desmoglein 1 beta	1	1.22	1
	ANXA2	12306	Annexin A2	3	12.98	3
	FN1	14268	Fibronectin	12	12.43	27
	HRG	94175	Histidine-rich glycoprotein	2	6.71	2
	VTN	22370	Vitronectin	5	17.15	15

**a -** Significant MS/MS number of peptides identified in blood EVs from CDAA-fed mice.

**b -** Significant MS/MS absolute % of coverage calculated in blood EV from CDAA-fed mice.

**c -** Significant MS/MS spectral counts identified in blood EVs from CDAA-fed mice.

**Figure 4:**
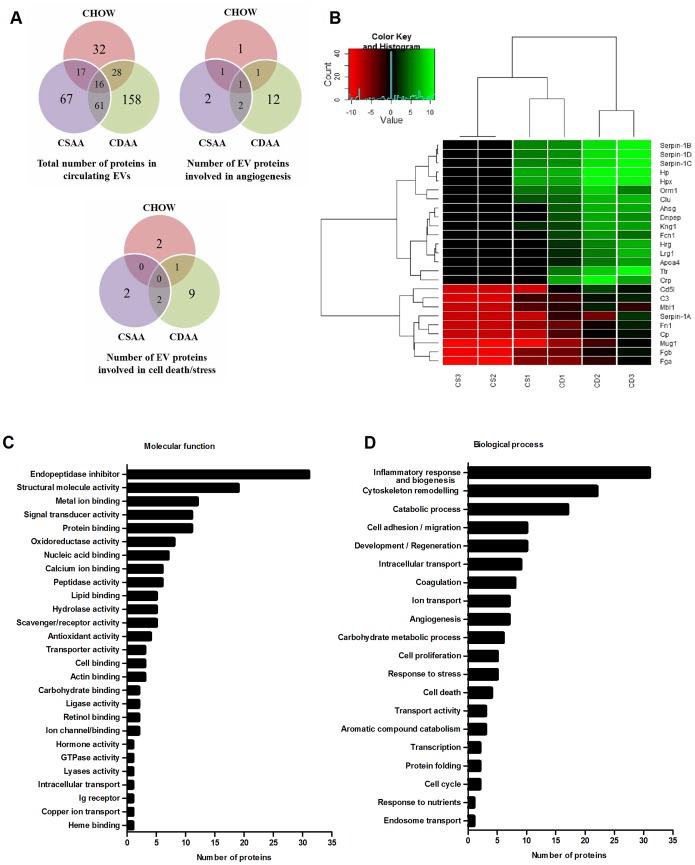
Overview on proteome changes of circulating extracellular vesicles in diet-induced NASH or control mice. (**A**) Venn diagrams depicting the total number of proteins and the number of proteins involved in cell death, stress and angiogenesis in EVs isolated from CDAA, CSAA or chow fed mice. (**B**) Heatmap and dendrogram based on log2 of normalized spectral counts for proteins elevated in EVs isolated from CDAA (n = 3, CD) compared to CSAA (n = 3, CS). Protein expression levels are color coded, showing a stronger and lower expression in green and red, respectively. Functional and molecular grouping of proteins identified in EVs isolated from CDAA-fed mice for 20 weeks. By using annotations derived from the Gene Ontology database, proteins have been organized based on (**C**) molecular function and (**D**) biological process.

Based on our proteomics analysis we found that several proteins were present in all three groups, but a significant number of proteins were present only in EVs isolated from CDAA-fed mice for 20 weeks (Fig. 4A). Additionally we observed that EVs isolated from CDAA-fed mice carry more proteins involved in NASH-associated outcomes such as cell death and stress and angiogenesis when compared with EVs from mice fed with CSAA or chow diet (Fig. 4A). In order to investigate whether the protein profile of blood EVs could be used to develop a signature that aids in discriminating NAFLD animals from controls, the total protein abundance for 158 targets were measured from 3 samples (different repeats from 3 groups of n = 4 mice) from the CSAA and CDAA diet groups and then normalized to the measurement from 12 normal chow mice. We then compared the abundance of each protein between the CDAA and CSAA group with the non-parametric two sample Wilcoxon (Mann-Whitney) test. 25 proteins with p-value <0.15 were considered candidate proteins for altered abundance. These 25 proteins were all up-regulated in CDAA diet mice (Table 2). The heatmap and dendrogram based on these 25 proteins show that the CDAA mice are clearly separated, and therefore distinct, from the CSAA mice (Fig. 4B). Findings show that within the most abundant proteins expressed in EVs isolated from CDAA-fed mice some are hepatocyte-specific and involved in one or more NAFLD-related outcomes, such as cell death, inflammation and pathological angiogenesis.

**Table 2:** A Nonparametric two-sample Wilcoxon test was performed in order to obtain a list of the most alternated proteins in EVs isolated from CDAA-fed mice for 20 weeks compared to EVs isolated from CSAA-fed mice.

Gene ID	Coverage (%)	Abbreviation	Description	Log_2_ ratio	W-test
20700	13.56	SERPIN-1A	Alpha-1-antitrypsin 1–1 (Serpin 1A)	9.19	0.064
20701	13.56	SERPIN-1B	Alpha-1-antitrypsin 1–2 (Serpin 1B)	5.31	0.077
20702	13.35	SERPIN-1C	Alpha-1-antitrypsin 1–3 (Serpin 1C)	5.61	0.077
20703	11.38	SERPIN-1D	Alpha-1-antitrypsin 1–4 (Serpin 1D)	5.54	0.077
15439	46.11	HP	Haptoglobin	4.75	0.077
15458	49.78	HPX	Hemopexin	4.61	0.077
11801	41.76	CD5L	CD5 antigen-like	10.56	0.064
18406	4.34	ORM1	Alpha-1-acid glycoprotein 1	3.87	0.077
12759	14.73	CLU	Clusterin	4.93	0.077
11625	24.06	AHSG	Alpha-2-HS-glycoprotein	6.84	0.064
13437	7.02	DNPEP	Aspartyl aminopeptidase	6.39	0.064
16644	4.37	KNG1	Kininogen-1	5.40	0.077
14133	2.69	FCN1	Ficolin-1	5.43	0.064
94175	6.71	HRG	Histidine-rich glycoprotein	6.36	0.064
76905	4.09	LRG1	Leucine-rich HEV glycoprotein	6.42	0.064
11808	4.55	APOA4	Apolipoprotein A-IV	5.76	0.064
22139	19.05	TTR	Transthyretin	9.79	0.064
12944	33.78	CRP	C-reactive protein	9.34	0.064
12266	33.61	C3	Complement C3	4.85	0.116
17194	15.16	MBL1	Mannose-binding lectin-1	5.21	0.077
14268	12.43	FN1	Fibronectin	7.53	0.077
12870	15.75	CP	Ceruloplasmin	8.48	0.064
17836	8.4	MUG1	Murinoglobulin-1	9.32	0.064
14161	16.7	FGA	Fibrinogen, A alpha polypeptide	5.66	0.116
110135	24.95	FGB	Fibrinogen, B beta polypeptide	6.30	0.077

Proteins identified in circulating EVs from CDAA fed mice for 20 weeks, could be assigned to Genome Ontology (GO) categories which are defined by the GO Consortium [37]. Based on the GO analysis of the newly discovered proteins, blood EVs carry cytosolic, extracellular, plasma membrane, and nuclear proteins. We found that some functional activities of oxidoreductase, hydrolase, endopeptidase inhibitors, signal transducers and lipid binding proteins were abundantly expressed in EVs, indicating that these activities might be of important relevance regarding the potential effect of the circulating EVs on the target cells or tissue (Fig. 4C). Moreover, the analysis identified a significant number of proteins involved particularly in inflammation and immunity, cell death, angiogenesis and cell adhesion, which represent all clinically-relevant features of NASH (Fig. 4D). These results suggest that circulating EVs carry a variety of different proteins, different in molecular function and biological processes that reflect the pathological progression of NASH.

### Circulating EVs from NAFLD animals contain liver-related microRNAs

**Figure 5:**
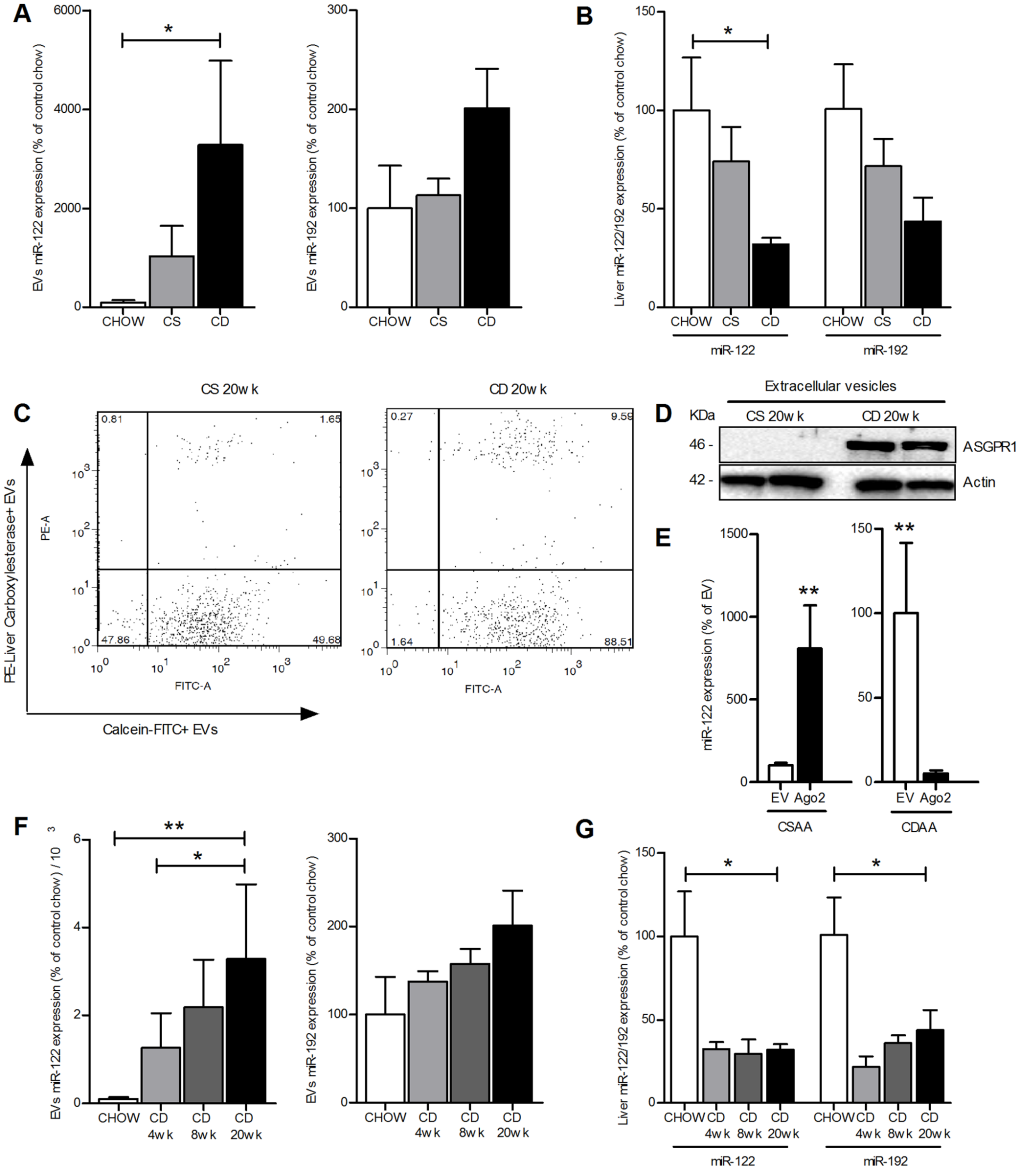
Identification of liver-related microRNAs encapsulated in EVs and released from liver during diet-induced NASH. (**A**) Expression levels of miR-122 and –192 in EVs and (**B**) liver isolated from CDAA, CSAA and chow fed mice for 20 weeks. (**C**) Analysis of Liver carboxylesterase-PE/Calcein-FITC positive circulating EVs isolated from CDAA and CSAA-fed mice for 20 weeks. (**D**) Western blot analysis of the hepatocyte-specific asialoglycoprotein-receptor (ASGPR1) in purified EVs isolated from CSAA and CDAA-fed mice for 20 weeks. (**E**) Expression level of miR-122 encapsulated in EVs or associated to Argonaute-2 (Ago2) in CSAA and CDAA-fed mice for 20 weeks. (**F**) Expression levels of miR-122 and –192 over time in circulating EVs and (**G**) liver harvested from CDAA-fed mice for 4, 8 or 20 weeks or control mice. Mean values were normalized to U6 snRNA. Values represent mean ± SD. *P<0.05, **P<0.005, ***P<0.0005, Kruskal-Wallis test with post-hoc Mann-Whitney test and Bonferroni correction.

**Figure 6:**
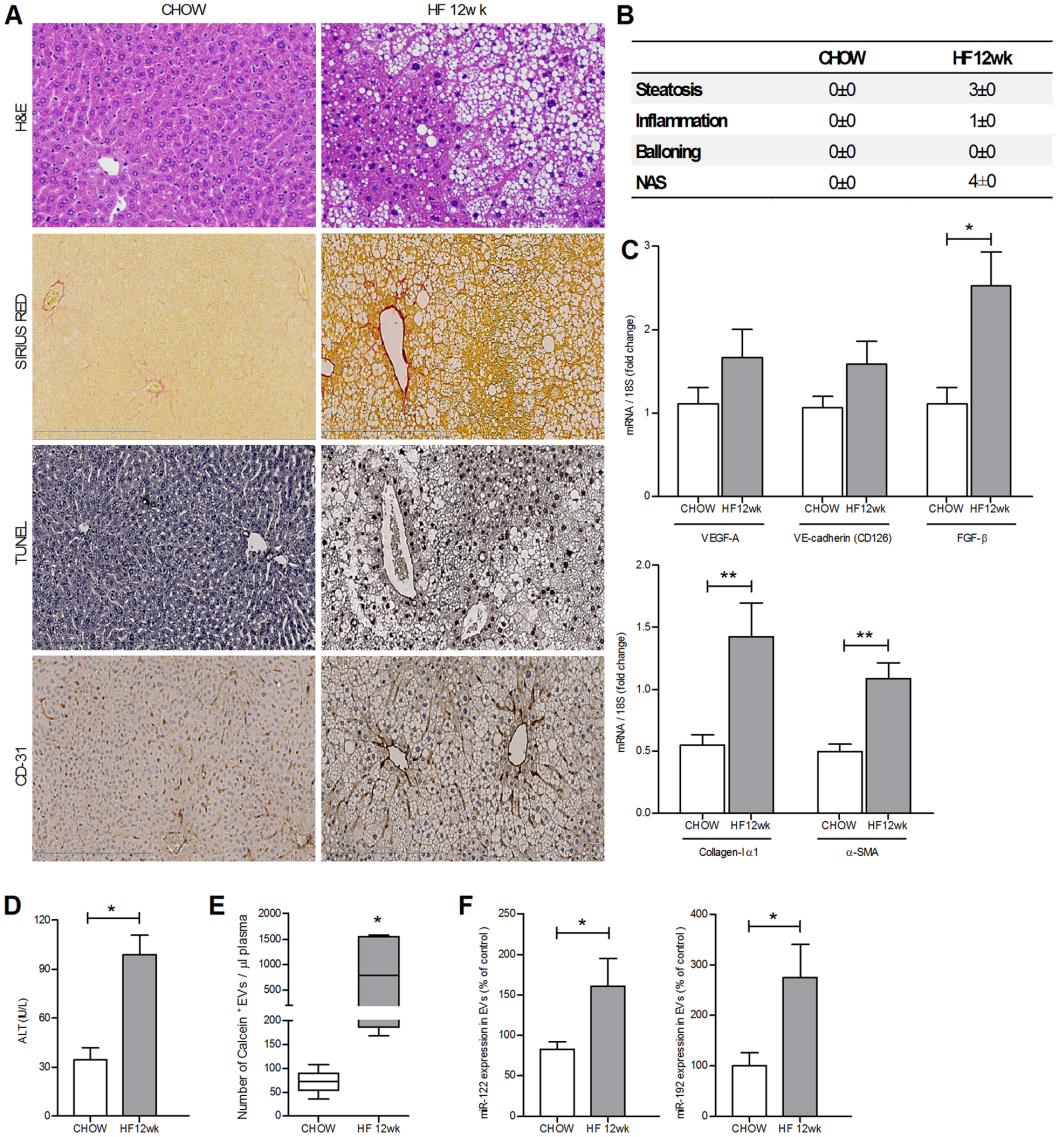
High fat diet induces liver steatosis and extracellular vesicles release in blood stream. (**A**) Liver specimens from wild type C57BL/6 mice (n = 6) fed with the high fat diet (HF) or chow diet for 12 weeks, were used for assessing liver damage (Hematoxylin & eosin staining, H&E), fibrosis by detecting the collagen deposition (Sirius red staining), cell death (TUNEL staining) and pathological angiogenesis (CD31 staining). (**B**) H&E liver specimens were analyzed in a blinded way for steatosis, inflammation and ballooning and NAS score was determined in mice fed with HF diet for 12 wks or with control diet.(**C**) Analysis of the expression of the transcripts for VEGF-A, FGF-β, CD126 (VE-cadherin), Collagen type-I, α-smooth muscle actin (α-SMA) and CTGF, assessed by quantitative real-time PCR. The housekeeping gene 18S was used as internal control. (**D**) Plasma alanine aminotransferase (ALT) levels were analyzed from mice fed with chow or high fat diet. (**E**) Whisker plot of flow cytometry analysis of circulating Calcein+ extracellular vesicles per µL of platelet-free plasma isolated from high fat (HF, n = 6) or chow diet fed mice (n = 6) for 12 weeks. (**F**) Expression levels of miR-122 and –192 in circulating EVs. Values represent mean ± SD. *P<0.05, **P<0.005, ***P<0.0005, Kruskal-Wallis test with post-hoc Mann-Whitney test and Bonferroni correction.

Various recent reports have provided evidence that EVs contain non-coding microRNAs from the cell of origin as part of their cargo and the presence of specific microRNAs can be used to identify the cell of origin [38], [39]. Moreover, we have recently demonstrated that hepatocytes release large quantities of EVs when exposed to lipotoxic fatty acids that have been shown to be important mediators of liver damage during NAFLD [40]. Based on these concepts we tested the hypothesis that hepatocytes might be an important source of circulating EVs in NAFLD animals. To test this hypothesis, we measured the level of miR-122 and miR-192, two microRNAs abundant in hepatocytes [41]–[43], in blood EVs from the CDAA treated and control mice. Indeed, we found that miR-122 and miR-192 levels in circulating EVs were significantly elevated in those isolated from CDAA-fed mice compared to those from mice on the control diet (Fig. 5A). As a consequence, and consistent to what has been recently reported in patients with severe NAFLD or NASH [44], miR-122 and miR-192 levels decreased in the livers of CDAA-fed mice for 20 weeks, compared to livers of mice fed with the control diets (Fig. 5B). Additionally, the amount of liver carboxylesterase (CES1) and hepatocyte-specific asialoglycoprotein-receptor (ASGPR1) positive EVs was significantly higher in CDAA-fed mice compared to mice fed with the control diet (1.65% vs. 9.59%) (Fig. 5C–D). While in healthy individuals, miR-122 has been shown to circulate almost exclusively in a non-membrane bound form associated with a specific protein, Argonaute2 (Ago2), while a recent report demonstrated that in NAFLD patients the majority of serum miR-122 circulates in Ago2-free forms [33]. Based on these findings and our results, we next tested the hypothesis that in experimental NASH miR-122 is primarily encapsulated in EVs. We therefore measured the level of miR-122 in the EV fraction and in the Ago2-linked fraction. We observed that miR-122 was mainly encapsulated in EVs in CDAA-fed mice for 20 weeks while it was primarily associated with Ago2 protein in mice fed with the control diet, where the presence of EVs is very limited (Fig. 5E). Furthermore, the levels of these two microRNAs increased in EVs from NAFLD animals over time (Fig. 5F). These findings, in conjunction with the findings of decreased levels of miR-122 and miR-192 in livers of mice fed with the CDAA diet compared to control mice (Fig. 5G), strongly suggest that damaged or stressed hepatocytes are a source of the increased circulating EVs and that hepatocyte-specific microRNAs are released through EVs in circulation over time during NAFLD progression. To evaluate whether these findings were related to liver injury associated with NASH, and not to the CDAA model per se, we used a second model of NAFLD by feeding mice with high fat (HF) diet for 12 weeks. Histopathological and gene expression analyses revealed that mice fed with the HF diet for 12 weeks developed NAFLD (Fig. 6A-6D). Importantly, as demonstrated by the CDAA model, the levels of circulating EVs were significantly elevated with HF feeding compared to chow (Fig. 6E). As shown in the CDAA model, the EVs isolated from HF mice displayed markedly up-regulated levels of miR-122 and miR-192 compared to EVs isolated from chow mice (Fig. 6F). Taken together these data strongly support the concept that EVs are generated during NASH development and are released in circulation potentially paving the way to the development of a new generation of mechanism-based biomarkers for NASH.

## Discussion

This study relates to the role of EVs as potential biomarkers of liver damage in NAFLD. Results demonstrate that diet-induced NAFLD/NASH is associated with marked increased production and release of EVs in the liver and in circulation. Detailed characterization of EVs identified both exosomes and microparticles released into the bloodstream during experimental NASH. Circulating EVs were enriched in miRNA-122 and miR-192 - two well-investigated and abundant microRNAs in hepatocytes. Moreover, blood EV levels were dynamic, increasing over time, becoming significantly higher as early as 8 weeks on the CDAA diet, a time point that is associated with early NAFLD, and closely correlating with key histopathological features of disease severity. A clustering analysis of the protein profile of blood EVs allowed for differentiation of NAFLD animals versus controls.

In light of the dramatic increase in the prevalence of NAFLD, and in conjunction with the significant research effort in developing novel therapies targeted to patients with NAFLD, dynamic and noninvasive biomarkers that can be measured periodically to track disease changes in real time, are in great need. Biomarkers would not only help in the diagnosis of NAFLD, but also be useful for the assessment of treatment response and prognosis. We hypothesized that EVs - membrane bound structures released from a variety of cell types during stress or apoptosis and have been increasingly linked to cell-cell communication - have the potential to fulfill these requirements. Two recent pilot studies in humans showed increased levels of leucocyte-derived MPs in patients with NAFLD and in patients with alcohol and/or chronic hepatitis C related cirrhosis supporting the concept that EVs are potential biomarkers to monitor liver injury [20]. Unfortunately, EVs derived from inflammatory cells are not specific and may be elevated in a number of immune and inflammatory conditions associated with their activation thus limiting their utility as biomarkers of liver disease [45]–[47]. Our data identify for the first time the proteome of circulating EVs from NAFLD animals and demonstrate that these vesicles carry a selective antigenic composition. Moreover, hierarchical clustering analysis allowed for discrimination of mice with established NASH from those with isolated steatosis, identifying for the first time a potential signature in blood EVs that might be used to diagnose NAFLD noninvasively. We further showed that blood EVs carry microRNAs and proteins (e.g. CES1) abundant in liver, strongly suggesting the liver as an important source of these EVs in circulation during NASH. Interestingly, we found a significant number of proteins involved in cell death (Cathepsin D, Clusterin, CD5 antigen-like), angiogenesis and cell-adhesion (Extracellular matrix protein 1, Desmoglein 1α/β, SHC-transforming protein 1, Annexin A2, Fibronectin, Galectin-3-binding protein, Vitronectin), antioxidant and redox signaling and inflammatory response processes. The enrichment of these proteins in circulating EVs suggests that they carry specific signatures, and materials, reflecting the pathological condition of the organism of origin. The presence of oxidative stress and production of reactive oxygen species (ROS) by dysfunctional mitochondria as a consequence of hepatocyte lipotoxicity during NAFLD progression [48], may explain the significant presence of proteins involved in redox signaling pathways and antioxidant enzymes. Importantly, we also detected a number of proteins noteworthy for their involvement in cell death, such as proteins that have serine/cysteine protease inhibitor domains [49]. We also identified the presence of a large number of proteins involved in pathological angiogenesis, including proteins responsible for cell motility, cell-cell adhesion, migration, sprouting and neovessel formation [50]. The proteome of circulating EVs of CDAA-fed mice also reflects the mechanisms of vesicle formation, as demonstrated by the presence of glycolytic enzymes, cytoskeleton structural proteins and GTPases, which play a role in the calcium-dependent or independent vesicle formation and trafficking processes. The presence of atherogenic lipoproteins and scavenger receptors may suggest a strong link between non-alcoholic steatohepatitis and cardiovascular complications, as have been previously described [51].

Based on these results, we further examined the potential role of the circulating extracellular vesicles, increased during NAFLD, for non-invasive monitoring of disease progression. The levels of circulating extracellular vesicles isolated from CDAA-fed mice for 4, 8 or 20 weeks were time-dependent, becoming significantly higher at the 8 week time point and strongly correlated with the histopathological features of NAFLD. In particular, the level of circulating EVs released during diet-induced NAFLD strongly correlated with hepatic fibrosis, cell death and pathological angiogenesis. Moreover, the circulating EVs from mice on the CDAA diet were enriched in miR-122 and miR-192, two abundant microRNAs in hepatocytes. The level of these microRNAs increased in EVs and decreased in livers over time during NAFLD progression. The release of these microRNAs from stressed or damaged hepatocytes in EVs during NAFLD progression may provide an attractive explanation for the decreased expression level of miR-122 found in the livers of patients with advanced NAFLD [44],as well as early stage hepato-carcinogenesis from NASH in both an animal model and human tissue samples, as recently reported [52]. Indeed, a recent study by Pirola et al [33] demonstrated that, as opposed to healthy individuals where miR-122 is present in circulation only in Ago2 complex fraction, in patients with NAFLD the majority of serum miR-122 circulates in Ago2-free forms. The specific compartment was not further assessed in this study and future studies to determine the circulating levels of miR-122 in EVs from patients with fatty liver disease are warranted.

In order to validate the results observed in the CDAA model of NAFLD/NASH, we studied a second murine model in which mice are fed a high caloric high fat diet for a total of 12 weeks. The similar results obtained with this model strongly suggest that the changes in blood EVs are related to NASH development and exclude a potential confounding effect of the CDAA diet. Thus, our findings show that EVs are produced and released during NAFLD progression, have a specific antigenic composition reflecting the pathological alterations typical of its progression and express microRNAs and proteins abundant in the liver. Additionally, the EV levels are dynamic and change over time, correlating with changes in liver histopathology characteristic of NAFLD/NASH. In conclusion, our study suggests that monitoring EVs in circulation may be a promising, novel, non-invasive tool to assess disease progression in NAFLD, and uncover the antigenic profile of these vesicles during experimental NAFLD development, which are potential targets for immune-based diagnostics development.

## Supporting Information

Figure S1 **Extracellular vesicles purification by sucrose gradient.** Extracellular vesicles in platelet-free plasma (PFP) samples isolated from CDAA- or CSAA-fed mice were purified by ultracentrifugation on a 10–70% sucrose-gradient to reduce contaminants and soluble proteins. (**A**) and (**B**) show the PFP samples separation after sucrose-gradient ultracentrifugation as detailed in Methods. (**C**) Silver staining gel of different fractions obtained after sucrose-gradient ultracentrifugation of PFP samples isolated from CDAA- and CSAA-fed mice for 20 weeks.
(TIF)

Table S1 **List of proteins in circulating extracellular vesicles isolated from CSAA-fed mice based on LC-MS/MS analysis**. Pure circulating extracellular vesicles were isolated from CSAA (control diet)-fed mice for 20 weeks and processed for proteomics analysis. Proteins are listed in the table with the corresponding gene symbol and description based on GO Consortium, number of peptides and percentage of coverage (% Cov). Proteomics data are representative of three independent experiments.
(DOCX)

Table S2 **List of primers used for the gene expression studies.**
(DOCX)
