# Supplementary figures and images for: Circulating Extracellular Vesicles with Specific Proteome and Liver MicroRNAs Are Potential Biomarkers for Liver Injury in Experimental Fatty Liver Disease
Source: PLoS One. 2014 Dec 3;9(12):e113651. doi: 10.1371/journal.pone.0113651 (PMC4254757; doi:10.1371/journal.pone.0113651)

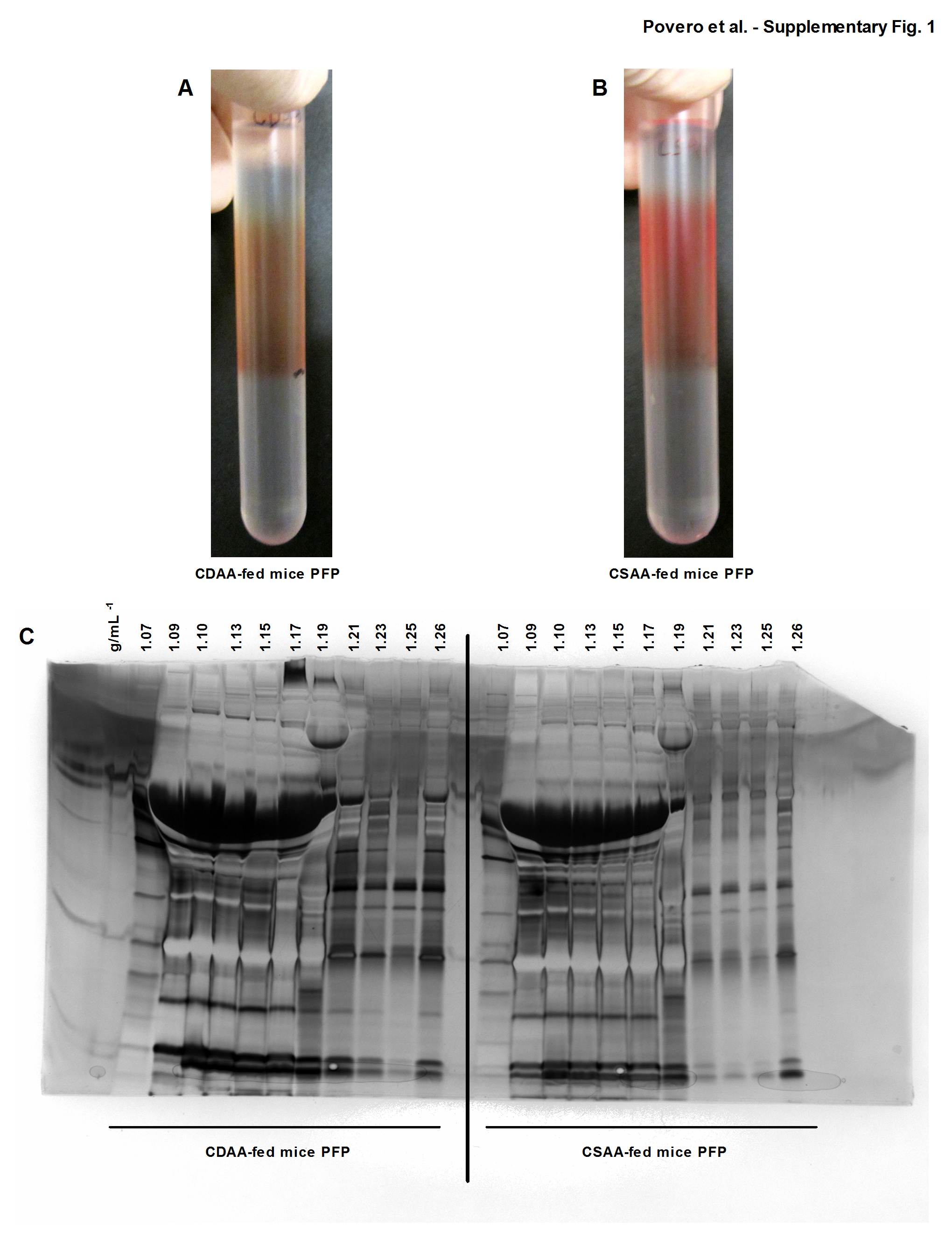

Supplement: Figure S1 — Extracellular vesicles purification by sucrose gradient. Extracellular vesicles in platelet-free plasma (PFP) samples isolated from CDAA- or CSAA-fed mice were purified by ultracentrifugation on a 10–70% sucrose-gradient to reduce contaminants and soluble proteins. (A) and (B) show the PFP samples separation after sucrose-gradient ultracentrifugation as detailed in Methods. (C) Silver staining gel of different fractions obtained after sucrose-gradient ultracentrifugation of PFP samples isolated from CDAA- and CSAA-fed mice for 20 weeks. (TIF) [file pone.0113651.s001.tif]
