# Supplementary material for: Circulating Extracellular Vesicles with Specific Proteome and Liver MicroRNAs Are Potential Biomarkers for Liver Injury in Experimental Fatty Liver Disease
Source: PLoS One. 2014 Dec 3;9(12):e113651. doi: 10.1371/journal.pone.0113651 (PMC4254757; doi:10.1371/journal.pone.0113651)
Supplement: Table S1 — List of proteins in circulating extracellular vesicles isolated from CSAA-fed mice based on LC-MS/MS analysis. Pure circulating extracellular vesicles were isolated from CSAA (control diet)-fed mice for 20 weeks and processed for proteomics analysis. Proteins are listed in the table with the corresponding gene symbol and description based on GO Consortium, number of peptides and percentage of coverage (% Cov). Proteomics data are representative of three independent experiments. (DOCX) [file pone.0113651.s002.docx]

| **Table S1.** Proteins identified by LC-MS/MS in circulating extracellular vesicles isolated from CSAA-fed mice | | | | |
| --- | --- | --- | --- | --- |
| **Gene symbol** | **Protein Description** | **Peptides (95%) ^a^** | **%Cov^b^** |  |
| Actg1 | Actin, gamma, 1 | 2 | 9.1 |  |
| Adipoq | Adiponectin | 1 | 4.85 |  |
| Alb | Albumin | 6 | 10.2 |  |
| Aldh1l1 | Cytosolic 10-formyltetrahydrofolate dehydrogenase | 6 | 13.75 |  |
| Alyref | THO complex subunit 4 | 1 | 7.1 |  |
| Ank1 | Ankyrin 1 | 13 | 15.69 |  |
| Apoa1 | Apolipoprotein A-I | 2 | 24.24 |  |
| Apoa2 | Apolipoprotein A-II | 1 | 9.8 |  |
| Apoe | Apolipoprotein E | 4 | 16.08 |  |
| Aqp1 | Aquaporin-1 | 2 | 10.78 |  |
| C1qa | Complement C1q, subcomponent subunit A | 10 | 44.9 |  |
| C1qb | Complement C1q subcomponent subunit B | 10 | 30.43 |  |
| C1qc | Complement C1q subcomponent subunit C | 9 | 21.54 |  |
| C1ra | Complement C1r, subcomponent A | 17 | 30.98 |  |
| C3 | Complement C3 | 39 | 31.63 |  |
| C4 | Complement C4 | 1 | 4.48 |  |
| Camp | Cathelin-related antimicrobial peptide | 2 | 25.43 |  |
| Clu | Clusterin | 3 | 10.49 |  |
| Colec11 | Collectin-11 isoform II | 1 | 6.98 |  |
| Cryab | Crystallin, alpha B | 3 | 27.43 |  |
| Dsp1 | Dual specificity phosphatase 19 | 1 | 0.76 |  |
| Epb41 | Erythrocyte protein band 4.1 | 2 | 7.71 |  |
| Epb42 | Erythrocyte protein band 4.2 | 3 | 13.89 |  |
| Fga | Fibrinogen, A alpha polypeptide | 6 | 22.08 |  |
| Fgb | Fibrinogen, B beta polypeptide | 7 | 23.08 |  |
| Fn1 | Fibronectin | 5 | 4.44 |  |
| Ftl1 | Ferritin | 3 | 26.78 |  |
| Glul | Glutamine synthetase | 1 | 7.5 |  |
| Gpx3 | Glutathione peroxidase 3 | 6 | 31.86 |  |
| Hbb-a1 | Alpha-globin 1 | 5 | 34.51 |  |
| Hbbt1 | Beta-globin | 10 | 74.15 |  |
| Hp | Haptoglobin | 11 | 37.18 |  |
| Hpx | Hemopexin | 10 | 36.09 |  |
| Igk | Immunoglobulin k chain | 33 | 43.4 |  |
| Jup | Desmoplakin | 4 | 8.32 |  |
| Kng1 | Kininogen-1 | 2 | 6.95 |  |
| Krt1 | Keratin 1, type II | 17 | 8.32 |  |
| Krt2 | Keratin 2 | 3 | 13.01 |  |
| Krt17 | Keratin 17, type I | 6 | 27.02 |  |
| Krt2 | Keratin 2, type II | 7 | 13.58 |  |
| Krt25 | Keratin 25, type I | 2 | 4.44 |  |
| Krtkb40 | Keratin kb40, type II | 1 | 3.56 |  |
| Krt5 | Keratin 5 | 8 | 15.52 |  |
| Krt6a | Keratin, 6A, type II | 14 | 41.31 |  |
| Krt6b | Keratin 6B, type II | 11 | 27.48 |  |
| Krt73 | Keratin 73, type II | 9 | 15.21 |  |
| Krt76 | Keratin 76 | 4 | 10.14 |  |
| Krt77 | Keratin 77 | 12 | 10.31 |  |
| Mbl1 | Mannose-binding lectin-1 | 3 | 16.8 |  |
| Mgam | Maltase-glucoamylase | 3 | 3.06 |  |
| Mvp | Major vault protein | 1 | 5.22 |  |
| Nes | Nestin | 1 | 2.73 |  |
| Orm1 | Alpha-1-acid glycoprotein 1 | 2 | 11.59 |  |
| Plak | Junction plakoglobin | 1 | 2.78 |  |
| Prdx2 | Peroxiredoxin-2 | 5 | 22.73 |  |
| Retnlg | Myeloid cysteine-rich protein | 4 | 47.01 |  |
| Serpin-1B | Alpha-1-antitrypsin 1-2 (Serpin 1B) | 7 | 20.34 |  |
| Serpin-1C | Alpha-1-antitrypsin 1-3 (Serpin 1C) | 6 | 13.59 |  |
| Serpin-1D | Alpha-1-antitrypsin 1-4 (Serpin 1D) | 6 | 11.62 |  |
| Serpin-1E | Alpha-1-antitrypsin 1-5 (Serpin 1E) | 5 | 11.14 |  |
| Serpin-K3 | Serine protease inhibitor A3K | 2 | 15.55 |  |
| Taok3 | Serine/threonine-protein kinase TAO3 | 1 | 2.28 |  |
| Tf | Serotransferrin | 34 | 46.92 |  |
| Tpm3 | Tropomyosin | 4 | 22.58 |  |
| Vcp | Transitional endoplasmic reticulum ATPase | 14 | 26.43 |  |
| Vtn | Vitronectin | 6 | 17.57 |  |
