# Supplementary material for: Circulating Extracellular Vesicles with Specific Proteome and Liver MicroRNAs Are Potential Biomarkers for Liver Injury in Experimental Fatty Liver Disease
Source: PLoS One. 2014 Dec 3;9(12):e113651. doi: 10.1371/journal.pone.0113651 (PMC4254757; doi:10.1371/journal.pone.0113651)
Supplement: Table S2 — List of primers used for the gene expression studies. (DOCX) [file pone.0113651.s003.docx]

**Table S2.** List of PCR primers used

| **Gene** | **Primers** |
| --- | --- |
| VE-cadherin | (FW) 5-CACTGCTTTGGGAGCCTTC-3  (RV) 5-GGGGCAGCGATTCATTTTTCT-3 |
| FGF-β | (FW) 5-TGTGTCTATCAAGGGAGTGTGT-3  (RV) 5-TGCCACATACCAACTGGAGTATT-3 |
| COL1A1 | (FW) 5-GCTCCTCTTAGGGGCCACT-3  (RV) 5-CCACGTCTCACCATTGGGG-3 |
| TIMP-1 | (FW) 5-CTTGGTTCCCTGGCGTACTC-3  (RV) 5-ACCTGATCCGTCCACAAACAG-3 |
| α-SMA | (FW) 5-GTCCCAGACATCAGGGAGTAA-3 (RV) 5-TCGGATACTTCAGCGTCAGGA-3 |
| CTGF | (FW) 5-GGGCCTCTTCTGCGATTTC-3  (RV) 5-ATCCAGGCAAGTGCATTGGTA-3 |
| VEGF-A | (FW) 5-GCACATAGAGAGAATGAGCTTCC-3 (RV) 5-CTCCGCTCTGAACAAGGCT-3 |
